# The impact of mobile-assisted swimming applications on intrinsic motivation and fear reduction in aquatic environments among students in the swimming course

**DOI:** 10.3389/fspor.2024.1496733

**Published:** 2024-12-05

**Authors:** Walaa Jumah Alkasasbeh, Thekra Alawamleh, Hasan Aloran, Tamara Farash, Bekir Erhan Orhan

**Affiliations:** ^1^ Program of Sports Management and Training, Department of Administration and Curriculum, Faculty of Arts and Educational Sciences, Middle East University, Amman, Jordan; ^2^ Department of Physical Education, Faculty of Sport Science, The University of Jordan, Amman, Jordan; ^3^ Faculty of Sports Sciences, Istanbul Aydın University, Istanbul, Türkiye

**Keywords:** mobile app, swimming applications, motivation, fear reduction, aquatic environments, swimming course

## Abstract

**Introduction:**

This study investigates the impact of mobile-assisted swimming applications on intrinsic motivation and fear reduction in aquatic environments among students enrolled in a swimming course. While technological tools are increasingly integrated into physical education settings, their effects on motivation and psychological barriers such as fear of water remain underexplored.

**Methods:**

A total of 69 male and female students enrolled in the “Swimming Education and Training” course participated in the study. The Intrinsic Motivation Scale (IMS) and the Water Fear Assessment Questionnaire (WFAQ) were used to measure intrinsic motivation and water-related fear, respectively, before and after using a mobile-assisted swimming application. Data were analyzed to assess changes in motivation and fear, and potential relationships with demographic variables (gender, academic year, parental swimming experience, and university GPA) were explored.

**Results:**

The study found no statistically significant differences in intrinsic motivation levels before and after the use of the mobile-assisted swimming application. However, a statistically significant reduction in water fear was observed following the intervention. No significant relationships were found between demographic factors and either motivation or fear reduction.

**Discussion:**

The results suggest that while the mobile-assisted swimming application did not significantly impact intrinsic motivation, it was effective in reducing fear of water among students. This highlights the potential of mobile applications in addressing psychological barriers in aquatic environments, supporting skill acquisition, and enhancing the overall learning experience in swimming courses. The absence of demographic influences suggests that the application's benefits may be broadly applicable across different student groups.

## Introduction

Swimming is a water sport that requires following rules and mastering a proper and efficient swimming technique (1, 2). Swimming encompasses any activity involving movement in water, such as walking or playing. It offers an enjoyable experience and a sense of contentment, allowing to make friends, compete, and experience a sense of accomplishment (3). Swimming also requires mastering the challenge of breathing correctly in water, which involves inhaling through the mouth and exhaling underwater (4). Psychological mechanisms play a significant role in influencing the effects of exercise.

Swimming is taught as an academic subject in numerous institutions, physical education programs, as well as health and sports programs (5). The educational process is characterized by ongoing and interactive engagement between students and instructors during educational activities (6). Within this process, there are two distinct types of learning activities: those performed by students and those facilitated by teachers. Both categories are conducted interactively, enhancing active interactions (7). During lectures, there is an elements in swimming education that supports students' ability to swim (8). These elements include mastering swimming techniques, understanding the theory of swimming education, and knowing how to apply the method when teaching swimming (9). Improving swimming learning can be achieved using a variety of supportive tools; these aids serve as effective means to assist in the learning process, regardless of its inherent drawbacks (10). Effective learning involves applying suitable teaching approaches and media to the subject presented by the teacher to the students. One example is the use of computers as an educational tool through multimedia learning (11).

Technology has evolved significantly in recent times, leading to a notable increase in the use of teaching resources in the technological world (12–14). This development is evident in the daily use of devices, games, and social media as primary tools for learning (15). During the COVID-19 pandemic, educators and students were expected to harness advanced technologies to enhance and innovate remote learning (16). The use of computers alongside innovative technology, including multimedia and the internet, facilitates a wide range of options for educators and students, fostering a heightened sense of purpose and interaction in the learning process (17).

Continuous technological advancements and the introduction of new mobile devices like smartphones and tablets have significantly enhanced convenience and practicality, making these devices indispensable in the lives of modern consumers (18). Modern technology enables mobile devices to be easily used for recording video feedback for athletes or students during physical education and sports training, even in settings like swimming pools (19).

The use of mobile media by students in the classroom is linked to teachers' confidence in their digital skills (20). Students are affected by teachers' views on technology in education, and their Intrinsic motivation to learn is affected by the presence of innovative teaching resources, such as mobile games, in schools (21). Intrinsic motivation is fueled by one's interests and curiosity, and innate satisfaction from engaging in an activity or learning experience (22). Individuals who are people who are intrinsically motivated frequently look for new information, are more self-driven and autonomous, and handle challenges more effectively (23). Intrinsic motivation better predicts long-term learning and performance compared to extrinsic factors like grades or rewards (24). Students with high intrinsic motivation in learning environments typically excel in challenging tasks, enjoy the learning process, engage deeply, and demonstrate creativity (25). Intrinsically motivated individuals engage in activities for enjoyment or personal challenge rather than responding to external stimuli or pressures (26). While the importance of intrinsic motivation may diminish with age, adults often retain a natural inclination toward intrinsic motivation (27). The university period is considered transformative, as individuals undergo significant changes (28). Ongoing training and professional growth should be provided for teachers in all fields, including training on specialized learning applications (14). Competent teachers are encouraged to use educational technologies, including mobile applications, to maximize their benefits (29). Learning to swim represents both a physical and cultural accomplishment (30). Consequently, many European countries incorporate swimming programs for beginners and advanced learners into their physical education curricula at different educational levels (31). There are numerous reasons why many people are unable to swim (32). These obstacles include limited access to swimming pools, cultural issues that result in a reluctance to learn swimming, racial factors such as hair care concerns, and discomfort with wearing swimwear. Parents’ fear of water can discourage their children from learning to swim, and injuries, drowning incidents, and negative experiences can also have an impact (33). However, the fear of drowning is a highly prevalent factor (32) and the strongest indicator of an inability to swim, even surpassing financial factors and access to swimming facilities (34). This fear can arise from a general fear of water (35). The fear of water, known as aquaphobia, is a type of “specific phobia” related to certain situations (36). The rate of this phobia ranges from 2% to 3% in the general populace (37). It is important for individuals with a fear of water, making it difficult to learn how to swim.Additionally, this fear should be identified and effective educational strategies developed to assist them (38). Psychological mechanisms play a significant role, especially in influencing the effects of exercise (39, 40).

Many students face difficulties in learning to swim due to fear of water (32, 33), which is a psychological barrier affecting their performance and motivation to learn swimming (34). With technological advancements and increased use of mobile devices, educational applications have emerged that can help reduce this fear and enhance students' motivation to learn swimming (19, 41).

This study aims to evaluate the impact of using mobile applications on students' motivation to learn swimming and the effectiveness of these applications in reducing fear of water among students in the College of Sports Sciences. The study focuses on addressing this significant issue and providing innovative educational solutions to improve the swimming learning process. It also aims to develop technology-based educational methods to enhance students’ academic and athletic performance. The study proposes the following hypotheses: First, the use of technology-assisted applications enhances students' motivation to learn swimming. Second, these applications contribute to reducing the level of students' fear of water.

## Methods and materials

### Methods

#### Intrinsic motivation scale

The researchers used the Intrinsic Motivation Scale (42) a 12-item assessment tool that employs a seven-point Likert scale to assess various factors, ranging from (1) does not agree at all to (7) agrees completely. The scores for intrinsic motivation range from (12 to 84), with higher scores indicating a higher level of intrinsic motivation. The IMS was tested on a sample of 20 students to verify its reliability and validity. The results indicated a Cronbach's alpha of 0.93, and the correlations between the individual items and the total scores showed statistical significance (*P* < 0.05), demonstrating the tool's reliability and validity, see Table 1.

**Table 1:** Reliability analysis of the intrinsic motivation scale.

Variable	Number of items	Cronbach's Alpha
Intrinsic motivation scale	12	0.93

#### Water fear assessment questionnaire (WFAQ)

We used a modified version of the Water Fear Assessment Questionnaire based on Misimi et al. (2020) (43). The original version had 20 questions, but one was removed, leaving 19 questions. One item related to the “river environment” was removed from the questionnaire to maintain relevance to the pool setting, which was the primary context of the study. However, questions addressing “open water” environments, including those referring to the sea and waves, were retained to assess participants' general water-related fears, which could extend beyond pool settings.This questionnaire includes Likert-type and multiple-choice items and takes about 10 min to complete. students used a five-point Likert scale to respond after visualizing themselves in a water scenario. Responses of “completely disagree” or “completely agree” were classified as “not afraid of water,” while scores of 4 or 5 indicated “fear of water.” With 19 questions, each scored from 1 to 5, the total score ranges from 19 to 95.

#### Study design

This study was conducted in the second semester of the 2023–2024 academic year (February) for students enrolled in the “Swimming Education and Training” course at a private university in Jordan. Students participated in practical swimming sessions twice a week, according to the schedule for the “Swimming Education and Training” course, Each session lasted one and a half hours.The study began by distributing two questionnaires to the students: the first to measure their Water Fear Assessment Questionnaire, and the second to assess their Intrinsic Motivation to learn. Ethical approval for the study was obtained from the Scientific Research Committee at the Faculty of Educational Sciences at Al-Ahliyya Amman university, in addition to obtaining the students' consent to participate in the study voluntarily and without any coercion. A quasi-experimental design was used, and the study sample consisted of 69 male and female students enrolled in the 'Swimming Education and Training' course, see Table 2. They were divided into two separate groups: one for males and one for females, due to the need for gender separation and the specific requirements of the course. The SwimtoFly® application was used as an innovative educational tool in this study to enhance swimming skills and increase users' confidence in the water. This application targets all skill levels, from beginners to competitors, whether they are children or adults. The app offers organized lessons that include rich educational content composed of carefully prepared videos by certified coach Christian Anseaume, who has extensive experience in teaching swimming. These lessons consist of five main educational units, including “Swimming with Confidence,” “Front Crawl,” “Backstroke,” “Butterfly,” and “Breaststroke,” allowing users the opportunity to learn both basic and advanced skills according to their different levels. As part of the study, students were asked to download the free SwimtoFly® version 1.5.1 app and were trained on how to use it. The app features four main options upon registration: “Swim Teacher,” “Swim Student,” “Triathlete or Competitor,” and “Parent.” When prompted by the app with the question “You are?”, students were directed to choose the “Swim Student” option. The app then asked them to specify their primary goal for learning to swim, offering multiple options including “Safety and Confidence,” “Stroke Techniques,” “Pleasure and Health,” and “Speed and Training.” Students were instructed to select “Speed and Training” as their primary goal. Additionally, the app features extra functionalities such as tracking progress and recording swimming distance and time spent swimming, which helps users continuously improve their performance. The effectiveness of the app has been enhanced by positive user experiences, as many parents have reported significant improvements in their children's confidence in the water and success in learning to swim, thanks to the interactive approach provided by the app. Thus, SwimtoFly® is considered an effective tool that supports the learning process in various swimming environments and contributes to the comprehensive development of swimming skills.

**Table 2:** Sample description.

Demographic variables		*N*	Percentage %
Sex	Male	35	50.72
	Female	34	49.28
Parents’ practice of swimming	Yes	18	26.09
	No	51	73.91
Academic year	First	5	7.25
	Second	17	24.64
	Third	14	20.29
	Fourth	33	47.83
University grade	Excellent	11	15.94
	Very good	40	57.97
	Good	17	24.64
	Satisfactory	1	1.45

One of the researchers involved in this study, who serves as a lecturer for the “Swimming Education and Training” course, monitored the students' use of the application and provided them with guidance and feedback during practical lessons based on the data recorded in the application. The study was conducted during the lectures throughout the entire semester, with the lecturer present during the training sessions on a continuous basis. During each session, the course lecturer reviewed students' performance through the application data, including distance covered and swim time, which helped him identify each student's strengths and weaknesses and provide precise feedback to improve their performance.

After observing the students' performance during training, the lecturer gave direct feedback to each student on the technical aspects needing improvement, such as refining their breathing technique or coordinating hand and leg movements. At the end of each session, the lecturer held group evaluation meetings to discuss the overall student performance, relying on the information recorded in the application. This enabled him to provide comprehensive feedback aimed at enhancing students' skills in upcoming exercises.

The lecturer also offered personalized guidance to some students based on their individual needs, referring to the video lessons available in the application as a tool to support skill improvement. The lecturer relied on the application data to evaluate students' overall progress throughout the semester, helping him monitor improvements in performance and reductions in water anxiety. This data enabled him to provide continuous feedback that contributed to enhancing students' learning and developing their swimming skills.

**Figure 1:**
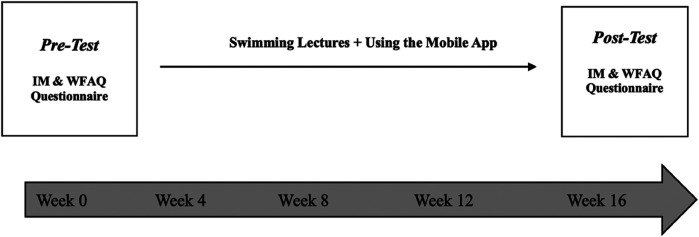
Study design.

At the end of the semester, researchers re-administered the questionnaires to the students to measure the development of their motivation to learn swimming and to determine whether the app had a psychological effect in reducing their fear of water and improving their swimming skills, see Figure 1.

#### Ethical considerations

This study adhered to the highest ethical standards in accordance with the guidelines established by the Scientific Research Committee at the Faculty of Educational Sciences at Al-Ahliyya Amman University. Ethical approval for the study was obtained prior to its commencement. All participants were fully informed of the study's purpose, procedures, and their rights as participants. Participation in the study was entirely voluntary, with no coercion or undue influence. Informed consent was obtained from all students, ensuring that they understood their involvement was optional and that they could withdraw from the study at any point without penalty or repercussions. Additionally, confidentiality was maintained throughout the study, with all data anonymized to protect the privacy and identity of participants.

#### Statistical analysis

The statistical processes in the study involved calculating means and standard deviations, followed by the application of paired-sample *t*-tests to compare pre- and post-intervention scores. Additionally, a two-way factorial ANOVA was used to assess the differences in application usage concerning motivation and fear of water among the study variables. The statistical analyses were conducted using SPSS software version 16.0.

## Results

Table 3 describes the differences in motivation levels and fear of water before and after using the mobile application, analyzed using a paired sample *t*-test. The results showed no significant differences in motivation levels before and after using the application, with scores of (55.8 ± 18.13) and (58.2 ± 13.06), respectively (*P* ≥ 0.05). In contrast, there were significant differences in fear of water, with scores of (3.04 ± 0.69) before and (2.82 ± 0.61) after using the application (*P* ≤ 0.05). In this context, higher motivation scores indicate better motivation, while lower fear scores indicate reduced fear, and the post-application results favored decreased fear of water.

**Table 3:** Pre- and post-intervention paired-sample *t*-test results for motivation and fear of water scores.

	Using the application	Mean ± std.	*n*	df	*t*	Sig.
Motivation	Pre	55.8 ± 18.13	69	68	−1.578	0.119
	post	58.2 ± 13.06				
Fear of water	Pre	3.04 ± 0.69	69	68	4.088	0.000
	post	2.82 ± 0.61				

Table 4 shows that the use of the application did not result in statistically significant differences in motivation levels or reduction in fear of water across various groups, including gender (males and females), whether parents practice swimming or not, academic year, or university grades. However, there were positive trends in motivation improvement and fear reduction, although these differences were not statistically significant (*P* ≥ 0.05).

**Table 4:** Differences in application Use for motivation and fear of water for study variables.

			Mean ± std.		*n*	*F*	Sig.
			Before using app	After using app			
Motivation	Sex	Male	54.17 ± 19.29	56.51 ± 15.96	35	.936	.337
		Female	57.50 ± 16.96	59.94 ± 9.095	34		
	Parents’ practice of swimming	Yes	56.83 ± 18.93	56.83 ± 13.85	18	.003	.953
		No	55.45 ± 18.02	58.69 ± 12.87	51		
	Academic Year	First & second	58.00 ± 20.16	57.64 ± 16.96	22	.195	.824
		Third	55.71 ± 15.68	54.00 ± 11.15	14		
		Fourth	54.39 ± 18.07	60.36 ± 10.50	33		
	University grade	Excellent	59.00 ± 18.91	57.91 ± 17.27	11	.450	.640
		Very good	57.08 ± 18.74	58.65 ± 12.97	40		
		Good & satisfactory	51.06 ± 16.26	57.39 ± 10.91	18		
Fear of water	Sex	Male	3.15 ± 0.72	2.97 ± 0.66	35	3.264	.075
		Female	2.92 ± 0.64	2.67 ± 0.51	34		
	Parents’ practice of swimming	Yes	3.25 ± 0.94	2.99 ± 0.91	18	2.354	.130
		No	2.95 ± 0.56	2.77 ± 0.46	51		
	Academic year	First & second	3.10 ± 0.68	2.89 ± 0.61	22	.923	.403
		Third	3.19 ± 0.96	2.95 ± 0.94	14		
		Fourth	2.93 ± 0.55	2.73 ± 0.40	33		
	University grade	Excellent	3.31 ± 0.73	2.90 ± 0.69	11	.682	.509
		Very good	3.03 ± 0.73	2.83 ± 0.66	40		
		Good & satisfactory	2.89 ± 0.52	2.77 ± 0.45	18		

## Discussion

The findings of this study provide essential insights into how mobile-assisted applications can impact intrinsic motivation and water fear among university students enrolled in swimming courses. While the results show clear potential for technology to reduce fear, its impact on motivation remains more complex and multifaceted. This section delves deeper into the underlying dynamics of the outcomes and explores the broader implications for educational practices and future research.The study hypothesized that the use of the mobile application would enhance intrinsic motivation in students learning swimming, a hypothesis grounded in previous research suggesting that digital tools, when applied in educational settings, have the potential to increase engagement and self-driven interest (44–46). The slight improvement in intrinsic motivation observed post-intervention, from a mean score of 55.8 to 58.2, while not statistically significant, raises important questions. One possible explanation is that intrinsic motivation in learning contexts is highly individualized and shaped by personal interests, past experiences, and the specific relevance of the task to the learner (47–49). For the groups, such as first-, second-, third and fourth-year students, the lack of significant change in motivation may imply that mobile technology alone does not provide sufficient stimulation for intrinsic motivation to flourish. The role of the instructor, peer dynamics, and students’ overall interest in the sport may play a more crucial role in shaping their intrinsic motivation than the mere presence of a technological tool (50, 51). Intrinsic motivation is strongly linked to self-efficacy, meaning students with low confidence in their swimming abilities may not have been sufficiently empowered by the app to experience meaningful improvements in motivation (52, 53). The lack of increased motivation likely stems from the app's inability to fully address the unique needs of those with lower self-efficacy, who may require more personalized support and feedback to feel capable of achieving success. Although the results were not statistically significant concerning intrinsic motivation, a positive trend towards reducing the fear of water was observed, with the mean fear score decreasing from 3.04 to 2.82, the results tend to agree with those of study (54), which found significant results, suggesting that exposure to controlled environments and repetitive learning scenarios may help reduce phobias and anxiety.The app used in this study provided structured guidance on swimming techniques, enabling students to rehearse movements, visualize their progress, and receive feedback, all factors that may contribute to improving swimming performance, reducing fear of water, and enhancing overall learning outcomes.This significant reduction was observed across various subgroups, including male and female students, students whose parents either practised or did not practice swimming, and students in their first, second, third and fourth years. This consistency in results suggests that the mobile application can serve as an effective educational intervention for fear reduction across diverse demographic categories (55, 56). By offering a safe, consistent, and accessible learning environment, mobile technology appears to alleviate one of the most critical psychological barriers to learning swimming: fear.

Interestingly, students from the first to the fourth year did not show a significant decrease in their fear of water, suggesting that academic progression may not be directly related to fear of water levels. Several factors could explain this finding. First, students’ fear of water might be more strongly influenced by their prior experiences rather than their academic year. The lack of significant differences between students from different years could also be attributed to the uniformity in the training course, the use of the same mobile application, and the consistent teaching methods employed, which may have limited the influence of academic progression on fear reduction. Moreover, academic advancement might not have a direct effect on emotional fears such as fear of water. While academic growth can enhance general confidence and knowledge acquisition, overcoming specific fears often requires more targeted interventions and psychological strategies. Research has shown that cognitive and emotional factors, such as psychological resilience and coping strategies, play a key role in how individuals respond to interventions aimed at reducing anxiety or fear, which may explain why certain students, regardless of their academic level, did not experience significant reductions in fear (57, 58). Furthermore, the study found no statistically significant relationship between university grade point average, gender, or parents' swimming practice and the level of fear of water or motivation to learn. This suggests that these demographic variables do not play a major role in influencing fear or motivation when using assistive applications. Academic performance, gender, and family swimming habits may not directly affect a student's ability to manage emotional fears, as personal experiences and direct exposure to training seem to be more influential in determining fear levels and motivation.

The use of mobile applications in education has been increasingly explored, particularly in physical education and sports contexts. This study contributes to this growing body of research by demonstrating that mobile apps can indeed be beneficial in reducing water-related fears, which are a significant barrier to learning to swim. Fear of water, or aquaphobia, is a common issue that can hinder many individuals from developing essential swimming skills (59–61). By reducing fear, students will likely feel more confident and capable of engaging with the water, leading to better learning outcomes. However, this study's limited impact on intrinsic motivation highlights a critical aspect of technology integration in education: technology alone cannot replace other key learning elements (62, 63). The role of instructors, peer support, and a conducive learning environment remains paramount. The mobile app can be viewed as a supplementary tool that facilitates certain aspects of learning, such as providing clear instructional videos, offering feedback, and allowing for repeated practice (64, 65). However, these tools may only significantly alter deeper motivational drives if coupled with broader pedagogical strategies that foster curiosity, self-efficacy, and personal relevance.

Moreover, the effectiveness of such applications may depend on their design and the extent to which they meaningfully engage learners (66, 67). While this study used a commercially available swimming app, future research might explore how custom-designed applications tailored specifically to student needs could more effectively foster intrinsic motivation. For example, apps that offer more personalized feedback, gamification elements, or social features might better engage students and lead to greater motivational outcomes.

The findings of this study have several practical implications for educators and curriculum developers. First, incorporating mobile-assisted learning tools in swimming education, especially for reducing water fear, appears to be an effective strategy to enhance students’ psychological comfort in aquatic environments (60, 61). Educators should consider integrating such technologies as part of a broader pedagogical approach, particularly for students who struggle with anxiety or phobias related to swimming. Second, the results suggest educators should not rely solely on technology to enhance intrinsic motivation. Motivation is a complex psychological construct that may require more holistic approaches, including personalized teaching methods, fostering student autonomy, providing meaningful feedback, and creating a supportive learning environment (56, 68). Mobile applications should be viewed as a larger puzzle in promoting student motivation and engagement (69, 70).

## Limitations and future research

This study has several limitations that should be acknowledged. One key limitation is its quasi-experimental design, which lacked a control group for direct comparison. While the pre-and pos*t*-test design provides valuable insights, future studies could benefit from randomized controlled trials to better isolate the effects of the mobile application. Additionally, the study was conducted over a single semester, which may not have been sufficient to observe more profound changes in intrinsic motivation. Longitudinal studies tracking students' progress over multiple semesters could provide a clearer picture of how motivation evolves with the continued use of mobile technology.

Another limitation is the reliance on self-reported measures for intrinsic motivation and fear of water. While these tools are considered valid and reliable, they may be subject to social desirability bias or inaccuracies in self-assessment. Incorporating objective measures, such as performance-based assessments of swimming skills, could provide a more comprehensive understanding of the app's impact.

A further limitation relates to the inclusion of questions addressing “open water” environments, such as the sea and waves, while excluding a question about “river environments.” This discrepancy may have influenced the lack of statistical significance in the ANOVA results, as participants might feel confident in pools but retain fears specific to open water environments. Future studies should consider using more context-specific questionnaires tailored to the primary aquatic setting being studied.

Finally, individual differences in technology adoption and learning outcomes were not explored. Factors such as prior experience with technology, baseline levels of motivation and anxiety, and personality traits could significantly influence students' responses to mobile-assisted learning tools. Customizing these tools to meet individual student needs might enhance their effectiveness in promoting motivation and fear reduction.

## Conclusion

In conclusion, this study highlights the potential of mobile-assisted swimming applications in significantly reducing water fear among Students in the Swimming Course. The findings emphasize the effectiveness of such applications in improving students' confidence and supporting swimming skill acquisition, particularly in environments where fear is a barrier to learning. However, the study also underscores the complexity of intrinsic motivation in educational settings, as no statistically significant improvements were observed in motivation levels. While mobile technology can play an essential role in the learning process, it cannot replace the need for personalized instruction and a supportive learning environment. Educators should consider integrating mobile applications as part of a blended learning approach that combines technological tools with traditional teaching methods. Future research should focus on the long-term effects of mobile-assisted applications on motivation and performance, exploring ways to customize these tools to meet the diverse needs of student populations. Overall, this study demonstrates the promise of mobile technology in physical education, while also highlighting the need for continued innovation and research in this rapidly evolving field.

## Data Availability

The original contributions presented in the study are included in the article/Supplementary Material, further inquiries can be directed to the corresponding author.
